# Supplementary figures and images for: Glycine soja, PI424025, is a valuable genetic resource to improve soybean seed-protein content and composition
Source: PLoS One. 2024 Nov 12;19(11):e0310544. doi: 10.1371/journal.pone.0310544 (PMC11556748; doi:10.1371/journal.pone.0310544)

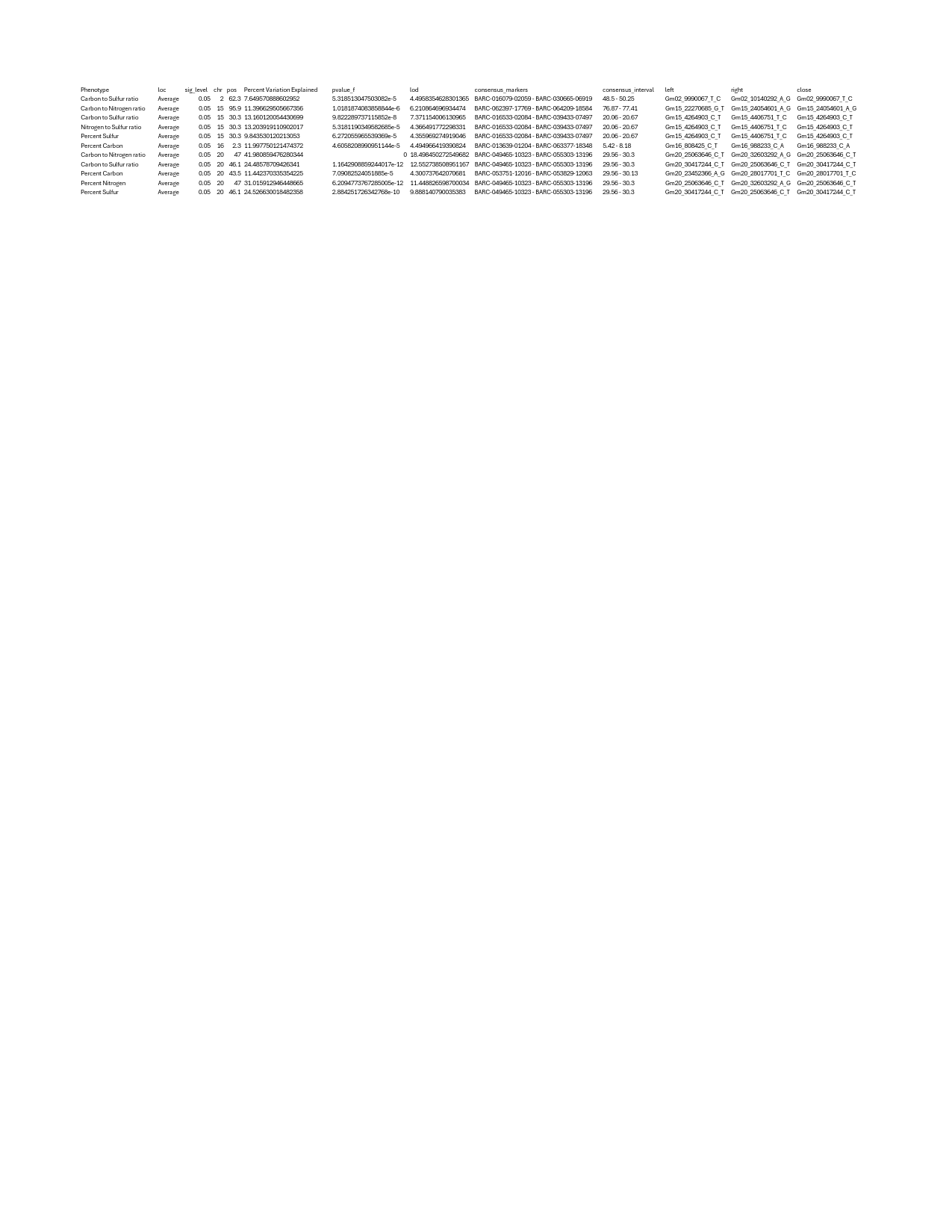

Supplement: S3 Data — The chromosome number, pvalue, lod, consensus markers, consensus interval, left marker, right marker and closest marker are reported. (TIFF) [file pone.0310544.s003.tiff]
